# Localization and in silico‐based functional analysis of miR‐202 in bull testis

**DOI:** 10.1111/rda.14159

**Published:** 2022-05-26

**Authors:** Bushra T. Mohammed, F. Xavier Donadeu

**Affiliations:** ^1^ Department of Pathology and Microbiology, College of Veterinary Medicine University of Duhok Duhok City Iraq; ^2^ The Roslin Institute and R(D)SVS University of Edinburgh, Easter Bush Roslin UK

**Keywords:** bull, in situ hybridization, miR‐202, testicular cells

## Abstract

Bull fertility is pivotal to the prosperity of the cattle industry worldwide. miR‐202 has been shown to be gonad specific and to have key roles in gonad function in different species. To further understand the involvement of miR‐202 in bull reproduction, this study aimed to establish its localization in bovine testicular tissue and to identify putative biological functions using bioinformatics approaches. We assessed the miR‐202 expression in paraffin‐embedded tissue samples collected form an abattoir using in situ hybridization. miR‐202 was present in Sertoli cells and in germ cells at different stages of development. Using available databases, a total of 466 predicted gene targets of miR‐202 were identified. Functional annotation revealed that miR‐202 target genes were mainly associated with protein modification and phosphorylation processes as well as longevity regulating pathway. Moreover, genes in the longevity regulating pathway mapped to PI3K/Akt/mTOR pathway which is involved in promoting proliferation of testicular cells and spermatogenesis. These findings suggest that miR‐202 plays important roles in regulating proliferation and viability of testicular cells including somatic and germ cells.

## 1 INTRODUCTION

Semen quality is an important determinant of reproductive success in cattle herds, whether AI or natural service are used. (Barth, 2018) Reduced bull fertility is a major cause of poor reproductive performance of herds worldwide. Studies indicate that 20%–40% of bulls are subfertile. (Khatun et al., 2013; Kastelic, 2013) Apart from economic implications, bull subfertilty leads to reduced animal welfare through an increased need for breeding and culling of repeat breeder cows. (Kastelic, 2013) Thus, a good understanding of the molecular mechanisms underlying testes function and spermatogenesis, and how these determine the production of high‐quality sperm, is essential to achieve high levels of productivity in the cattle industry. (Taylor et al., 2018; Rexroad et al., 2019)

Spermatogenesis is a highly organized process within the seminiferous tubules in the testes. Sertoli cells and Leydig cells play a pivotal role in the initiation and maintenance of sperm development as well as in regulation of male hormone production. (Phillips et al., 2010) Spermatogenesis involves three main events, spermatocytogenesis, meiosis and spermiogenesis. During spermatocytogenesis, germ cells differentiate and give rise to spermatogonial stem cells, which actively undergo mitotic division to generate two set of diploid primary spermatocytes. These then undergo meiosis I to produce two haploid secondary spermatocytes. Each haploid secondary spermatocyte differentiates into two haploid spermatids via meiotic cell division II, resulting in production of four haploid spermatids. During spermiogenesis, spermatids differentiate and become spermatozoa which migrate into the lumen of seminiferous tubules in a process called spermiation. (Staub & Johnson, 2018; Valli et al., 2015)

Testicular development and function are tightly regulated by microRNAs (miRNAs), which act by modulating the expression of a wide range of protein‐coding genes involved in cell differentiation processes within the male reproductive system.(Barbu et al., 2021; Fernández‐Pérez et al., 2018; Papaioannou, 2010) Mammalian testes express a large set of miRNAs including several tissue‐specific sequences.(Gao et al., 2020; Rakoczy et al., 2013; Yang et al., 2013) Expression of the gonad‐specific miRNA, miR‐202, is highly conserved across species including mouse, human, bovine, boars, chicken, Zebra fish and salamander.(Wainwright et al., 2013; Dabaja et al., 2015; Bannister et al., 2011; Chen et al., 2017; Presslauer et al., 2017; Sontakke et al., 2014; Revay et al., 2015) miR‐202 is expressed in the testicular somatic cell compartment(Chen et al., 2017) as well as in germ cells at different stages of development.(Chen et al., 2017; Jia et al., 2015) Studies showed that miR‐202 was localized in Sertoli cells of mouse testes and that it mediated some of the effects of the testis‐determining factor SOX9, involved in early gonad development.(Wainwright et al., 2013) A recent study showed that miR‐202 was robustly expressed in Sertoli cells of fertile men but was absent in sterile men.(Dabaja et al., 2015) Moreover, Chen et al showed that miR‐202 was expressed at high levels in mouse spermatogonial stem cells, and that CRISPR‐Cas9‐mediated miR‐202 knockout resulted in premature cell differentiation with loss of stemness, as well as increased mitosis and apoptosis.(Chen et al., 2017) As regards to bovine, a recent study showed that miR‐202 was highly enriched in sperm, and that sperm‐borne miRNA regulates the first cleavage in bovine embryos.(Wang et al., 2021) The aim of this study was to determine the expression pattern of miR‐202 in the bull testes, as well as to identify broader potential roles of this miRNA by using in silico target prediction.

## 2 METHODS

### 2.1 In situ hybridization

Testes from three healthy, 9‐month‐old bulls were obtained at an abattoir and transported in phosphate‐buffered saline (PBS) at 4°C within an hour of collection. Once in the laboratory, the testes were dissected into small pieces and fixed in 4% PFA treated with diethylpyrocarbonate (DEPC) (Sigma‐Aldrich,). After overnight fixation, tissue sections were cut at 6 μm and denatured with 5 μg/ml of proteinase K in 75 ml PBS then fixed in 4% PFA for 10 min and rinsed with 0.2% Glycine in PBS. The tissues were incubated with freshly prepared imidazole buffer then slides were placed in a humid chamber and freshly prepared 1‐ethyl‐3‐(3‐dimethylaminopropyl) carbodiimide (EDC) was added to each slide for 1 hr at RT followed by 2 hr of pre‐hybridization with 50% formamide and 5x SSC buffer at 25°C. Sections were then incubated overnight with Double Digoxigenin labelled LNA modified oligonucleotide probes (Exiqon,) against either bta‐miR‐202 (100% homologous sequence to human miR‐202‐5p; 80 nM), U6snRNA (positive control, 3 nM) or a scrambled sequence (negative control, 80 nM) in hybridization buffer. After the application of probe, slides were covered with gel bond film and heated to 60°C for 5 min, then placed in humidifying chamber at 50°C. After overnight incubation, slides were sequentially washed with 4x, 2x and 0.2x SSC post‐hybridization buffer for 10 min at 50°C to avoid unspecific binding and then rinsed with 1x Tris‐buffered saline (TBS). Slides were then incubated with blocking solution for 1 hr at RT. Anti‐digoxigenin antibody (1:200) was added to slides for 2 hr at RT followed by colour development with NBT/BCIP at 4°C for up to 16 hr. The signal was analysed with a light microscope. Independent analyses were performed using three different testicular sections from each animal.

### 2.2 Target prediction and gene enrichment analysis

miRNA target prediction was performed using three different algorithms, miRMap (v1.1), TargetScan 7.2 and miRWalk (v3.0). (Agarwal et al., 2015; Vejnar & Zdobnov, 2012) ClueGO (v2.5.7) plus Cluepedia (v1.5.7) software were used to identify Gene Ontology (GO) terms and Kyoto Encyclopedia of Gene and Genome (KEGG) pathways. ClueGO enables analysis of gene sets from organisms including bovine and considers many identifier types subtracted from a variety of sources including NCBI, UniPrtKB and Ensembl. (Bindea et al., 2009) Two‐sided hypergeometric tests were used for enrichment analyses, Benjamini–Hochberg correction was used for *p* value correction and Kappa coefficient of 0.4 was used to indicate the resemblance of GO terms for associated genes. The resulting GO terms with *p* < .01 and KEGG pathways with *p* < .05 were considered significant. Furthermore, the results were visualized using Cytoscape (v3.8.2).

## 3 RESULTS

### 3.1 Localization of miR‐202 in bull testes

**FIGURE 1:**
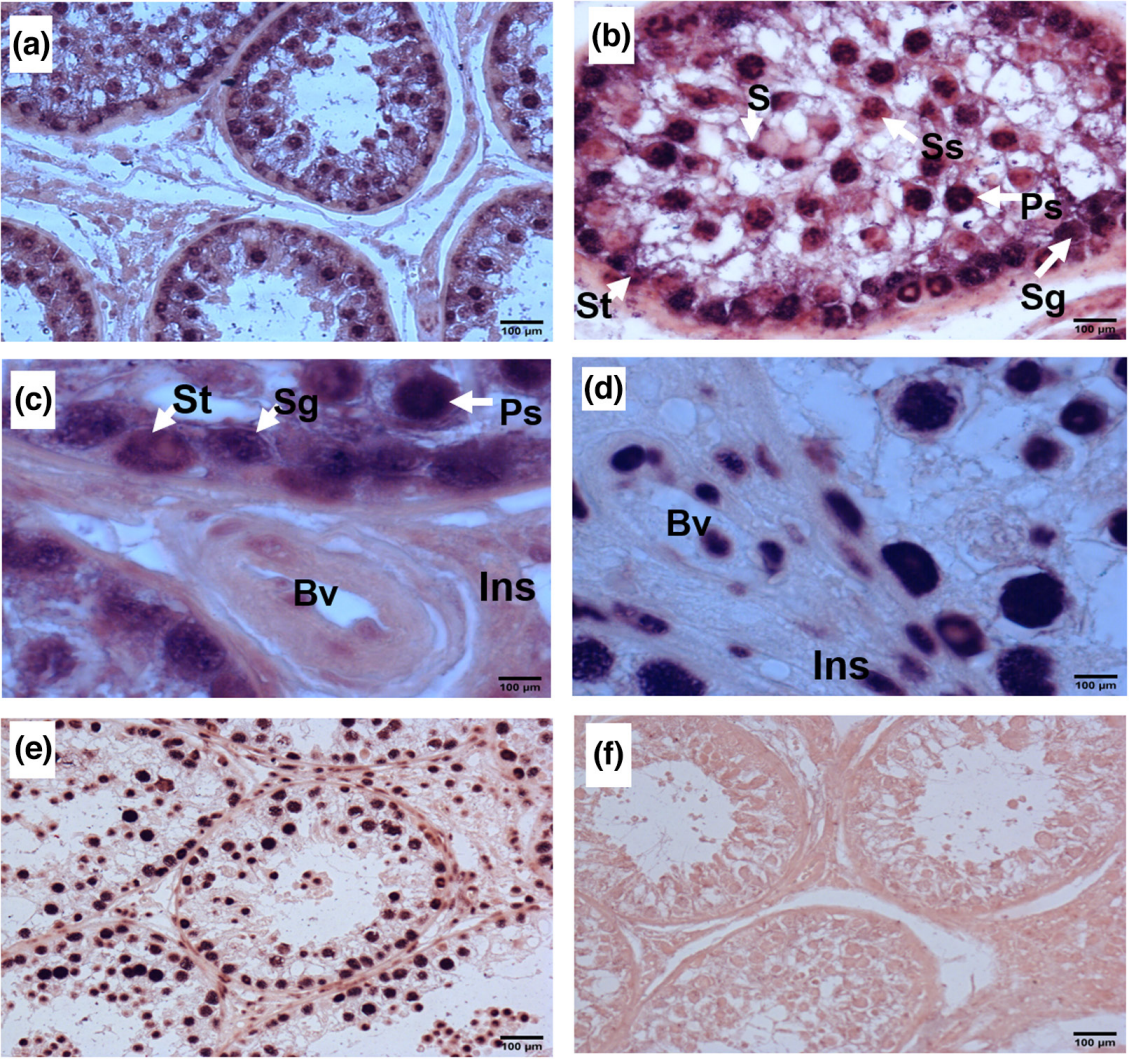
Representative images of in situ hybridization detection of miR‐202 in sections of bull testes (*n* = 3 animals). Sertoli cells (St), spermatogonia (sg), primary spermatocytes (Ps), secondary spermatocytes (ss) and spermatids (S) are indicated by white arrows. Blood vessels (Bv) and interstitial cells (ins) are also shown. Sections hybridized with probes against miR‐202 (a, b, c), U6B (d, e) and scrambled sequence controls (f) are shown. Original magnification 200x,400x and 1,000x. Scale bar, 100 μm

Testes from three bulls were examined using ISH to establish the cellular location of miR‐202. Different testicular cell types were identified based on position, size and shape. miR‐202 was detected in both Sertoli and spermatogenic cells (Figure 1a,b). Staining of miR‐202 was most pronounced in the cytoplasm of Sertoli cells, whereas staining in spermatogenic cells was localized mostly to the nucleus (Figure 1c). In germ cells, miR‐202 signal was particularly strong in spermatogonia and primary spermatocytes located near the basal compartment of the seminiferous tubule, as compared to secondary spermatocyte and spermatids (Figure 1b). These results suggest changes in miR‐202 expression at different developmental stages during spermatogenesis. No miR‐202 signal was detected in blood vessels and interstitial cells (Figure 1c). In addition, whereas the positive control, RnU6, displayed a strong nuclear localization in all sections (Figure.1d,e), the negative control showed no signal (Figure 1f).

### 3.2 miRNA target prediction and enrichment functional analysis

**FIGURE 2:**
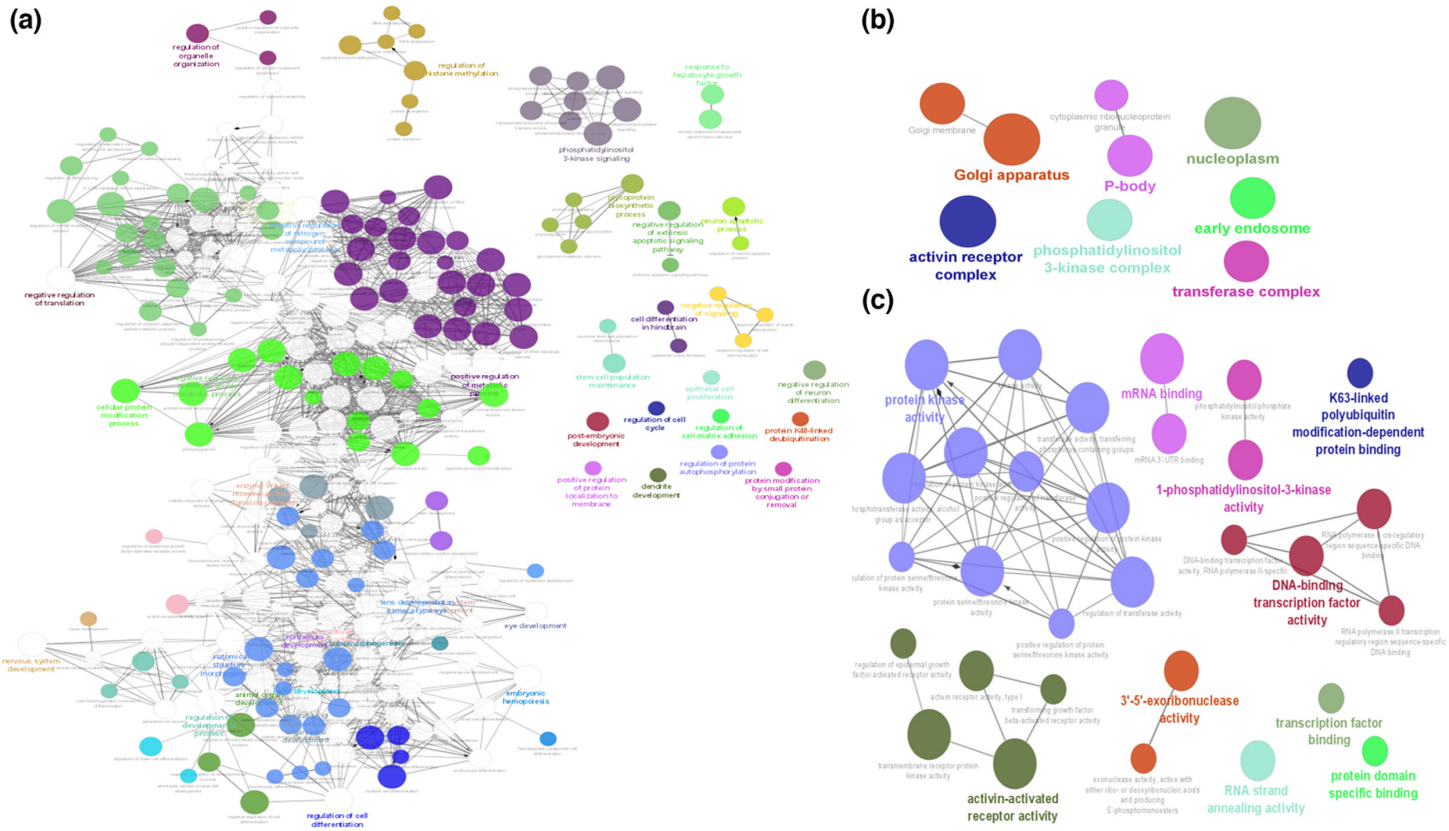
Functional GO terms enriched for target genes of miR‐202 identified using ClueGO and CluePedia (*p* ≤ .01). Different functional groups are represented by colours. Each node represents a GO term, and node size represents level of significance for term enrichment. Terms are connected based on shared genes. Enriched biological processes (a), cellular components (b) and molecular functions (c) of miR‐202 targets

**FIGURE 3:**
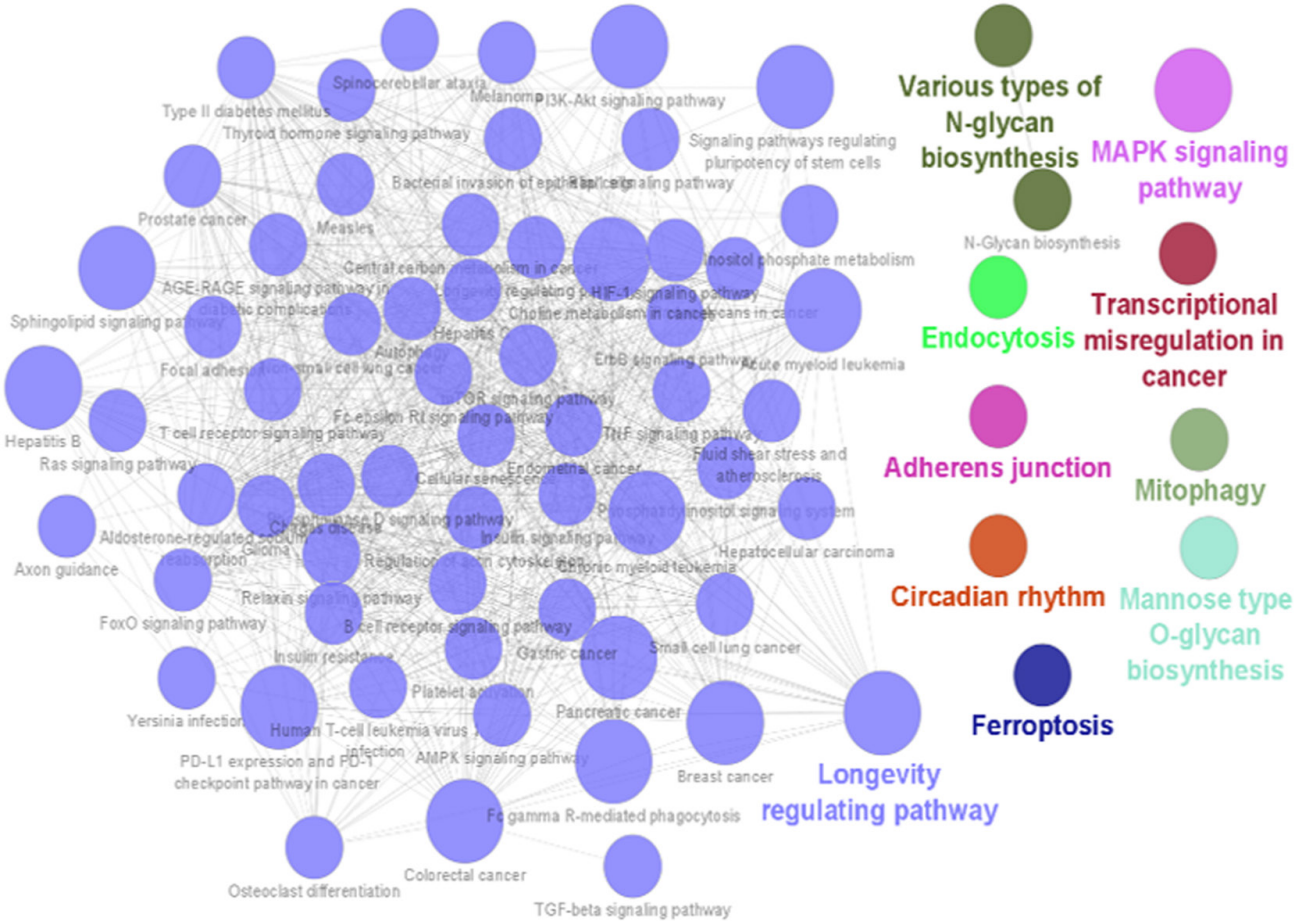
Functional KEGG pathways enriched for target genes of miR‐202 identified using ClueGO and CluePedia (*p* ≤ .05). Different functional groups are represented by colours. Each node represents a GO term, and node size represents level of significance for term enrichment. Pathways are connected based on shared genes

In order to gain insight into the roles of bta‐miR‐202 in bovine testis, miRNA target prediction tools were used. A total of 466 target genes were identified using miRMap, TargetScan 7.0 and miRWalk databases (Table S1). Gene ontology (GO) analysis using all identified targets showed that target genes were significantly enriched for several biological processes (BP), cellular components (CC) and molecular functions (MF) (Figure 2a–c). The most significant GO terms were protein modification process (GO:0036211, *p* < 1.09E‐08), nucleoplasm (GO:0005654, *p* < 5.04E‐07) and phosphotransferase activity, alcohol group as acceptor (GO:0016773, *p* < 4.49E‐05), in BP, CC and MF categories respectively. Kyoto Encyclopedia of Genes and Genomics (KEGG) pathway enrichment showed a subset of significantly enriched pathways for bta‐miR‐202 target genes, the most significant of which was the longevity regulating pathway (KEGG:04211, *p* < .0043) (Figure 3). This pathway includes genes such as CREB1, EIF4E, PIK3CA, PIK3CB, PIK3R1, PRKAA1, RB1CC1 and RPS6KB1 which are involved in PI3K/Akt/mTOR signalling.

## 4 DISCUSSION

The primary aim of the present study was to characterize the expression pattern of miR‐202‐5p in the bovine testis. The results indicate cell‐type‐dependent expression of miR‐202 during spermatogenesis. miR‐202 was localized in the cytosol of Sertoli cells, consistent with its involvement in post‐transcriptional target gene regulation in those cells. In humans, miR‐202‐5p was highly enriched in Sertoli cells, its expression differed between fertile and sterile men, and a role was suggested in mediating the interaction between somatic and germ cells during spermatogenesis. (Dabaja et al., 2015) A different study reported that miR‐202‐5p was highly expressed in Sertoli cells in mouse and chicken embryonic gonads. (Bannister et al., 2011; Wainwright et al., 2013) We also showed that miR‐202 was present in germ cells at all stages of development. In agreement with this finding, miR‐202‐5p was expressed throughout spermatogenesis in Medaka, demonstrating its involvement in male gamete development and differentiation. (Qiu et al., 2018) Another study demonstrated that miR‐202‐5p was significantly enriched in spermatozoa and developing male germ cells at different stages in zebrafish, (Jia et al., 2015) all together suggesting a conserved role in male germ cell function.

Our analyses identified ‘protein‐modification process’ as a top predicted function of miR‐202 target genes. In addition, the target genes of bta‐miR‐202 were significantly enriched in several molecular functions (GO terms) related to phosphotransferase and kinase activity which have a role in phosphorylation of many signalling proteins that are involved in regulation of mitochondrial activity, motility and apoptosis of testicular cells. (Gervasi & Visconti, 2017; Jankovičová et al., 2018; Silva et al., 2015) Furthermore, the top significant KEGG term enriched for miR‐202 target genes was longevity‐regulating pathway. The genes in the longevity regulatory pathway, including CREB1, EIF4E, PIK3CA, PIK3CB, PIK3R1, PRKAA1, RB1CC1 and RPS6KB1 are associated with several signalling pathways including PI3K/Akt/mTOR signalling. (Salas‐Pérez et al., 2019) PI3K/Akt /mTOR pathway is implicated in many cellular processes such as cell growth, survival, metabolism and autophagy. (Deng et al., 2021) The proliferation of Sertoli cells, which play a key role during spermatogenesis by facilitating adjacent germ cells with access to nutrients and growth factors, (Deng et al., 2021; Kimmins et al., 2004) is stimulated by FSH signalling through PI3k/Akt/mTOR. (Riera et al., 2012) Moreover, hyperactivation of phosphatidylinositol 3‐kinase (PI3K)/Akt/mTOR signalling is linked to different forms of cancer including testicular cancer. (Xu et al., 2017)

## 5 CONCLUSION

Our findings demonstrate that miR‐202 is expressed in Sertoli cells and, at varying levels, in different developmental stages of germ cells in bull testes. We also provide evidence suggesting the involvement of miR‐202 in multiple protein regulation, metabolism and longevity regulating pathways in the testes. Overall, these findings are consistent with critical roles of miR‐202 in regulating maturation and viability of testicular somatic and germ cells in bull testes.

## AUTHOR CONTRIBUTIONS

BTM and FXD contributed to the study conception and design. Methods and data analysis were performed by BTM. BTM and FXD drafted the manuscript and all authors read and approved the final manuscript.

## CONFLICT OF INTEREST

The authors declare that they have no conflict of interests.

## Supporting information


Appendix S1

## Data Availability

The datasets used and/or analyzed during the current study are available from the corresponding author on reasonable request.
